# A pre-symptomatic incubation model for precision strategies of screening, quarantine, and isolation based on imported COVID-19 cases in Taiwan

**DOI:** 10.1038/s41598-022-09863-w

**Published:** 2022-04-11

**Authors:** Grace Hsiao-Hsuan Jen, Amy Ming-Fang Yen, Chen-Yang Hsu, Sam Li-Sheng Chen, Tony Hsiu-Hsi Chen

**Affiliations:** 1 School of Oral Hygiene, College of Oral Medicine, Taipei Medical University, Taipei, Taiwan; 2 Institute of Epidemiology and Preventive Medicine, College of Public Health, National Taiwan University, Taipei, Taiwan; 3 Department of Emergency, Dachung Hospital, Miaoli, Taiwan

**Keywords:** Diseases, Mathematics and computing

## Abstract

Facing the emerging COVID viral variants and the uneven distribution of vaccine worldwide, imported pre-symptomatic COVID-19 cases play a pivotal role in border control strategies. A stochastic disease process and computer simulation experiments with Bayesian underpinning was therefore developed to model pre-symptomatic disease progression during incubation period on which we were based to provide precision strategies for containing the resultant epidemic caused by imported COVID-19 cases. We then applied the proposed model to data on 1051 imported COVID-19 cases among inbound passengers to Taiwan between March 2020 and April 2021. The overall daily rate (per 100,000) of pre-symptomatic COVID-19 cases was estimated as 106 (95% credible interval (CrI): 95–117) in March–June 2020, fell to 37 (95% CrI: 28–47) in July–September 2020 (p < 0.0001), resurged to 141 (95% CrI: 118–164) in October–December 2020 (p < 0.0001), and declined to 90 (95% CrI: 73–108) in January–April 2021 (p = 0.0004). Given the median dwelling time, over 82% cases would progress from pre-symptomatic to symptomatic phase in 5-day quarantine. The time required for quarantine given two real-time polymerase chain reaction (RT-PCR) tests depends on the risk of departing countries, testing and quarantine strategies, and whether the passengers have vaccine jabs. Our proposed four-compartment stochastic process and computer simulation experiments design underpinning Bayesian MCMC algorithm facilitated the development of precision strategies for imported COVID-19 cases.

## Introduction

As of 31st May 2021, coronavirus disease 2019 (COVID-19) pandemic has resulted in over 170 million cases and claimed 3.55 million lives after more than two large epidemic waves that had emerged between March and June 2020 and then resurged from October 2020 until May 2021^1^. It is well understood that most of these re-surging epidemics in the wake of the antecedent epidemic that has been already contained after the implementation of non-pharmaceutical interventions (NPIs) were originated from the spread of those imported COVID-19 cases. As imported COVID-19 cases play an important role in the global spread of COVID-19, all strategies of border control (including screening, quarantine, and isolation) of inbound and outbound passengers are therefore urgently needed before the full uptake of effective vaccine worldwide.

However, there is a trade-off between border control for containing COVID-19 and re-opening travel to resume pre-pandemic social and economic activities. One of solutions is to provide precision strategies of border control based on screening measures together with quarantine and isolation days as short as possible for travelers at international travel points of entry to each country or region. It is even important to provide precision strategies of border control for vaccinated and unvaccinated travelers^2,3^.

Of all transmission modes, the pre-symptomatic mode and the asymptomatic mode may play a more important role in border control of imported COVID-19 cases compared with the symptomatic mode because those who are symptomatic at the points of entry would be isolated but those asymptomatic and pre-symptomatic cases can only be detected through real-time polymerase chain reaction (RT-PCR) test or in quarantine until the presence of symptom for those pre-symptomatic cases. Ferretti et al. estimated that the contribution to the epidemic in China 2020 included 46% from pre-symptomatic cases, 38% from symptomatic cases, 10% from asymptomatic cases, and 6% from environmentally medicated transmission via contamination^4^. However, a series of meta-analyses have shown the heterogeneity of the proportion of asymptomatic cases ranging from 8.44% (95% CI: 5.12–13.62%) to 48% (95% CI: 30–67%)^5–10^, which may be a reflection of geographic variation with respect to test strategies, the dominance of severe acute respiratory syndrome coronavirus 2 (SARS-CoV-2) variants, and coverage rate of vaccination. Estimation of the distribution of different transmission modes considering the geospatial variation and the recent emerging variant of concern is needed when the global travelling is re-opened and precision strategies are provided for those under the suspicion of asymptomatic and pre-symptomatic cases during border control^11^.

The gold standard of evaluating these border control strategies is to conduct a series of randomized controlled trials (RCTs) in the domain of public health. However, it is impracticable for the emergency use during COVID-19 pandemic and also for delicate precision strategies designed by the conventional RCTs^12,13^. The alternative resorts to modelling the natural history of COVID-19 in the absence of strategies and then making use of computer simulation experiments on precision strategies, both of which are pertaining to two key parameters, the chance of being pre-symptomatic after the exposure to infective abroad calibrated by the risk-stratified countries or regions and the dwelling time from pre-symptomatic to the presence of symptoms, making allowance for one ancillary parameter, the daily rate of being asymptomatic (detected with RT-PCR) given close contacts with symptomatic cases. The chance of being pre-symptomatic and asymptomatic measures the risk level of contagiousness of each region/county. The dwelling time for pre-symptomatic cases determines the optimum time required for quarantine. The attempt made to consider the heterogeneity across countries and regions, the period, and the coverage and the efficacy of vaccination administered in each country or region can further facilitate precision quarantine and isolation policy. In addition, given the fast evolution of variants of concern (VOC) that have different behaviors, such as higher transmissibility like Omicron or higher virulence like Delta^14,15^, a natural history model of COVID-19 using data from different epoch with varying dominant VOCs can further facilitate the adjustment for the quarantine and isolation policy.

We propose a pre-symptomatic incubation model to estimate the natural history from the occurrence of being pre-symptomatic and the subsequent progression from pre-symptomatic to symptomatic phase with the parameters directly estimated with data on imported cases flown into Taiwan between March 2020 and April 2021, covering four phases, the first period (March–June 2020), the second period (July–September 2020), the third period (October–December 2020), and the fourth period (January–April 2021). The country/region-dependent incidence rate and dwelling time were directly estimated. Based on these two parameters by period and country or region, computer simulation experiments were envisaged and conducted to provide precision strategies for the frequencies of RT-PCR test and the optimal interval of quarantine and isolation for vaccinated and unvaccinated travelers in the face of the imported viral variants.

## Results

### Descriptive results

There were a total of 757,453 arrivals between March 2020 and April 2021, of whom we ascertained 290 symptomatic cases upon arrival, 283 pre-symptomatic cases during 14-day self-quarantine and self-isolation and 478 asymptomatic cases. The frequencies of three types of imported cases among the passengers flown to Taiwan from different areas in March–June, July–September, October–December 2020, and January–April 2021 around the world are detailed in Supplementary Table S1.

### Disease progression for COVID-19

The overall daily rate (per 100,000) of being pre-symptomatic during RT-PCR detectable phase was 106 (95% CrI (credible interval): 95–117; interquartile range (IQR): 102–110) in the first period, fell to 37 (95% CrI: 28–47; IQR: 34–40) in the second period (p < 0.0001), resurged to 141 (95% CrI: 118–164; IQR: 132–149) in the third period (p < 0.0001), and declined to 90 (95% CrI: 73–108; IQR: 84–96) in the fourth period (p = 0.0004) (Table 1). The corresponding median dwelling times were 3.44 (95% CrI: 3.09–3.82; IQR: 3.64–3.85), 4.26 (95% CrI: 2.94–6.00; IQR: 3.71–4.73), 4.01 (95% CrI: 3.27–4.86; IQR: 3.73–4.28), and 4.44 (95% CrI: 3.65–5.36; IQR: 4.14–4.73) days.

**Table 1:** The estimated daily rate of being pre-symptomatic and asymptomatic, and progression from pre-symptomatic to symptomatic COVID-19 among Taiwanese imported cases.

	Daily risk of being pre-symptomatic (per 10^5^) (95% CrI)	Median time to develop symptoms (Days) (95% CrI)	Proportion of asymptomatic case (%) (95% CrI)
March 2020–April 2021	101 (94–109)	3.64 (3.17–4.14)	26.5 (23.4–29.7)
**By period**			
March–June 2020	106 (95–117)	3.44 (3.09–3.82)	16.7 (13.3–20.5)
July–September 2020	37 (28–47)	4.26 (2.94–6.00)	28.5 (19.0–38.8)
October–December 2020	141 (118–164)	4.01 (3.27–4.86)	30.4 (22.1–39.4)
January–April 2021	90 (73–108)	4.44 (3.65–5.36)	47.8 (40.2–55.9)
**UK and USA in October–December 2020**			
UK	357 (164–617)	6.04 (3.32–9.86)	30.3 (10.4–55.7)
USA	283 (194–384)	3.24 (2.45–4.13)	22.0 (10.9–36.1)
**UK and USA in January–April 2021**			
UK	123 (39–244)	5.02 (1.93–10.09)	55.1 (28.1–81.0)
USA	157 (101–227)	3.61 (2.53–4.91)	44.4 (30.0–59.5)
**Vaccinated travelers from UK**			
	42 (8–127)	–	65.5 (24.5–93.2)

Vaccinated travelers from UK: The vaccine efficacy was applied to the scenario in the UK in October–December 2020.

The lower bottom panel of Table 1 shows the corresponding figures of all estimates for the USA and the UK, and the UK for the vaccinated travelers. Regarding the vaccinated travelers, the daily incidence rate (per 100,000) of pre-symptomatic cases was reduced to 42 (95% CrI: 8–127; IQR: 21–53), which renders the proportion of asymptomatic cases soar up to 65.5% (95% CrI: 24.5–93.2%; IQR: 52.6–80.3%) as the pre-symptomatic and symptomatic cases were substantially reduced by vaccination. The corresponding proportions of asymptomatic cases among those who showed no signs and symptoms increased from 16.7% (95% CrI: 13.3–20.5%; IQR: 15.4–17.9%) in the first period, 28.5% (95% CrI: 19.0–38.8%; IQR: 25.0–31.9%) in the second period (p = 0.0102), 30.4% (95% CrI: 22.1–39.4%; IQR: 27.5–33.5%) in the third period (p < 0.0001), and 47.8% (95% CrI: 40.2–55.9%; IQR: 45.0–50.5%) in the fourth period (p < 0.0001).

**Figure 1:**
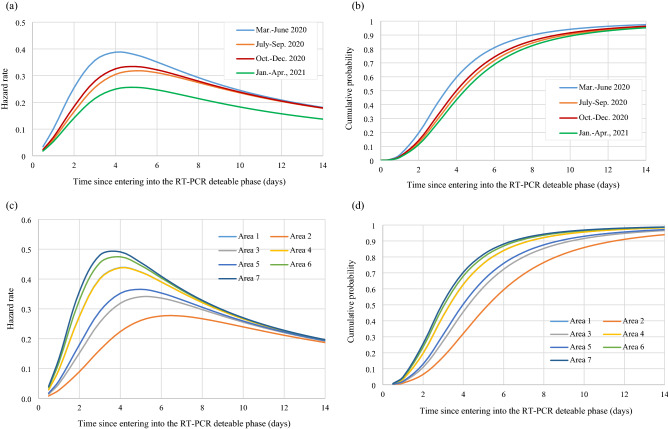
Clinical characteristics of COVID-19 of developing symptoms for pre-symptomatic cases of COVID-19. (**a**) Hazard rate (**b**) Cumulative probability of developing symptoms (**c**) Hazard rate by area during March and June 2020 (**d**) Cumulative probability of developing symptoms by area during March and June 2020, Taiwan; Area 1: Asia; Area 2: Oceania; Area 3: North and South America, excluding the U.S.; Area 4: U.S.; Area 5: Europe, excluding the U.K.; Area 6: U.K.; Area 7: Africa.

Figure 1a shows the instantaneous potential of progression from pre-symptomatic to symptomatic phase with time (in day), which increased with time until days 3–4 after being infected and decreased afterwards. It was estimated that around 80.9% and 90.0% of pre-symptomatic cases in the first phase would develop symptoms in 5 and 7 days, respectively (Fig. 1b). These findings suggest the scheduled time required for quarantine and isolation. The corresponding figures were 71.7% and 84.3% for the second period, 74.2% and 85.9% for the third period, and 68.8% and 82.4% for the fourth period. About 98% pre-symptomatic cases turning into symptomatic phase would be expected during a 14-day quarantine irrespective of any period.

For the time to develop symptoms among pre-symptomatic cases, the higher the incidence rate, the shorter the dwelling time. The hazard rate and cumulative probabilities of developing symptoms from pre-symptomatic phase by areas are delineated in Fig. 1c,d. The estimated results by areas are provided in Supplementary Table S2.

### Results of three-arm computer simulation experiments

By three risk groups defined by the daily rate of being pre-symptomatic (low- [< 300 per 100,000], intermediate- [300–500 per 100,000], and high-risk [> 500 per 100,000] group) during the first period (March–June), the simulated results based on the estimated parameters are shown in Table 2. These yielded the numbers of unidentified pre-symptomatic and symptomatic COVID-19 cases for three policies in the light of computer simulation experiments rolled shown in Supplementary Fig. S1, assuming 95% and 80% sensitivity of first and second of RT-PCR test. The Arm 1 following two RT-PCR tests with 5-day quarantine and isolation would not find any COVID-19 case regardless of any risk group and the periods, and the scenario for the vaccinated travelers from the UK. The corresponding figures for unidentified pre-symptomatic and asymptomatic COVID-19 cases were 6.3 (including 1.3, 1.7, and 3.3 from low-, intermediate-, and high-risk group, respectively) and 10.6 (including 0.6, 4.6, and 5.4 from low-, intermediate-, and high-risk group, respectively) for the second policy following one RT-PCR test with 5-day quarantine and isolation in the first period of March–June. The corresponding figures for the third period of October–December 2020 were 3.0 and 4.5 for the UK and 0.4 and 1.9 for the USA, respectively. With 50.6% and 43.3% population receiving at least one dose of vaccine in the UK and USA in January–April 2021, the number was reduced to 0.8 and 3.4 for the UK and 0.3 and 2.9 for the USA. However, pre-symptomatic and asymptomatic cases would be further reduced to 0.4 and 2.1 if only vaccinated travelers from the UK is considered. The third policy following 14-day self-quarantine and self-isolation without RT-PCR test exhibited 15.1 (including 4.2, 3.9, and 7.0 from low-, intermediate-, and high-risk group, respectively) pre-symptomatic cases and 212.6 (including 12.2, 91.9, and 108.5 from low-, intermediate-, and high-risk group, respectively) asymptomatic cases. The corresponding figures for the third period of October–December 2020 were 12.5 and 90.2 for the UK and 0.8 and 37.6 for the USA, respectively. In the fourth period of January–April 2021, the number was further reduced to 3.4 and 68.4 in the UK and 0.7 and 57.2 in the USA. Pre-symptomatic and asymptomatic cases were reduced to 1.5 and 41.7 for the vaccinated travelers from the UK. The similar findings were noted for the requirement of 7 and 14 days of quarantine.

**Table 2:** Simulated number of missed cases of imported passengers in the randomized controlled design during March and June in Taiwan using Markov Chain Monte Carlo methods

Strategy	Missed cases of imported COVID-19	March–June 2020 by risk of the departing countries			October–December 2020		January–April 2021		UK, vaccinated
		Low-risk	Intermediate-risk	High-risk	UK	USA	UK	USA	
**Arm1: Two RT-PCRs + 5-d Q (Sensitivity: 95% and 80%)**									
	Pre-symptomatic	0.3 (0.1–0.6)	0.3 (0.2–0.5)	0.7 (0.3–1.4)	0.6 (0.1–1.5)	0.1 (0.0–0.2)	0.2 (0.0–0.6)	0.1 (0.0–0.2)	0.1 (0.0–0.3)
	Asymptomatic	0.1 (0.0–0.3)	0.9 (0.6–1.2)	1.1 (0.3–2.6)	0.9 (0.3–1.8)	0.4 (0.2–0.6)	0.7 (0.3–1.3)	0.6 (0.3–0.9)	0.4 (0.1–1.2)
**Arm1-1: Two RT-PCRs + 7-d Q (Sensitivity: 95% and 80%)**									
	Pre-symptomatic	0.2 (0.0–0.4)	0.2 (0.1–0.3)	0.3 (0.1–0.7)	0.4 (0.1–1.1)	0.0 (0.0–0.1)	0.1 (0.0–0.4)	0.0 (0.0–0.1)	0.1 (0.0–0.4)
	Asymptomatic	0.1 (0.0–0.3)	0.9 (0.6–1.2)	1.1 (0.3–2.6)	0.9 (0.3–1.8)	0.4 (0.2–0.6)	0.7 (0.3–1.3)	0.6 (0.3–0.9)	0.4 (0.1–1.2)
**Arm1-2: Two RT-PCRs + 14-d Q (Sensitivity: 95% and 80%)**									
	Pre-symptomatic	0.0 (0.0–0.2)	0.0 (0.0–0.1)	0.1 (0.0–0.2)	0.1 (0.0–0.4)	0.0 (0.0–0.0)	0.0 (0.0–0.2)	0.0 (0.0–0.0)	0.0 (0.0–0.1)
	Asymptomatic	0.1 (0.0–0.3)	0.9 (0.6–1.2)	1.1 (0.3–2.6)	0.9 (0.3–1.8)	0.4 (0.2–0.6)	0.7 (0.3–1.3)	0.6 (0.3–0.9)	0.4 (0.1–1.2)
**Arm2: One RT-PCR + 5-d Q (Sensitivity: 95%)**									
	Pre-symptomatic	1.3 (0.3–3.2)	1.7 (1.1–2.6)	3.3 (1.3–6.8)	3.0 (0.7–7.4)	0.4 (0.1–0.8)	0.8 (0.0–2.7)	0.3 (0.1–0.8)	0.4 (0.0–1.3)
	Asymptomatic	0.6 (0.2–1.5)	4.6 (3.2–6.1)	5.4 (1.6–13.2)	4.5 (1.6–8.8)	1.9 (0.8–3.2)	3.4 (1.4–6.6)	2.9 (1.7–4.4)	2.1 (0.5–5.9)
**Arm2-1: One RT-PCR + 7-d Q (Sensitivity: 95%)**									
	Pre-symptomatic	0.8 (0.2–2.2)	0.9 (0.5–1.5)	1.7 (0.6–3.7)	2.1 (0.4–5.6)	0.2 (0.1–0.4)	0.5 (0.0–2.2)	0.2 (0.0–0.4)	0.3 (0.0–1.0)
	Asymptomatic	0.6 (0.2–1.5)	4.6 (3.2–6.1)	5.4 (1.6–13.2)	4.5 (1.6–8.8)	1.9 (0.8–3.2)	3.4 (1.4–6.6)	2.9 (1.7–4.4)	2.1 (0.5–5.9)
**Arm2-2: One RT-PCR + 14-d Q (Sensitivity: 95%)**									
	Pre-symptomatic	0.2 (0.0–0.8)	0.2 (0.1–0.3)	0.4 (0.1–0.8)	0.6 (0.1–2.1)	0.0 (0.0–0.1)	0.2 (0.0–1.0)	0.0 (0.0–0.1)	0.1 (0.0–0.3)
	Asymptomatic	0.6 (0.2–1.5)	4.6 (3.2–6.1)	5.4 (1.6–13.2)	4.5 (1.6–8.8)	1.9 (0.8–3.2)	3.4 (1.4–6.6)	2.9 (1.7–4.4)	2.1 (0.5–5.9)
**Arm3: 14-d Q**									
	Pre-symptomatic	4.2 (0.6–15.0)	3.9 (1.9–6.9)	7.0 (2.2–16.2)	12.5 (1.5–41.2)	0.8 (0.2–1.9)	3.4 (0.1–19.9)	0.7 (0.1–2.0)	1.5 (0.1–6.6)
	Asymptomatic	12.2 (3.5–30.9)	91.9 (64.7–123.0)	108.5 (32.4–263.3)	90.2 (32.9–176.6)	37.6 (16.8–64.3)	68.4 (27.8–132.2)	57.2 (34.9–87.2)	42.2 (10.2–118.5)

x-d Q: x-day quarantine; UK, vaccinated: The vaccine efficacy was applied to the scenario in the UK in October–December 2020.

**Figure 2:**
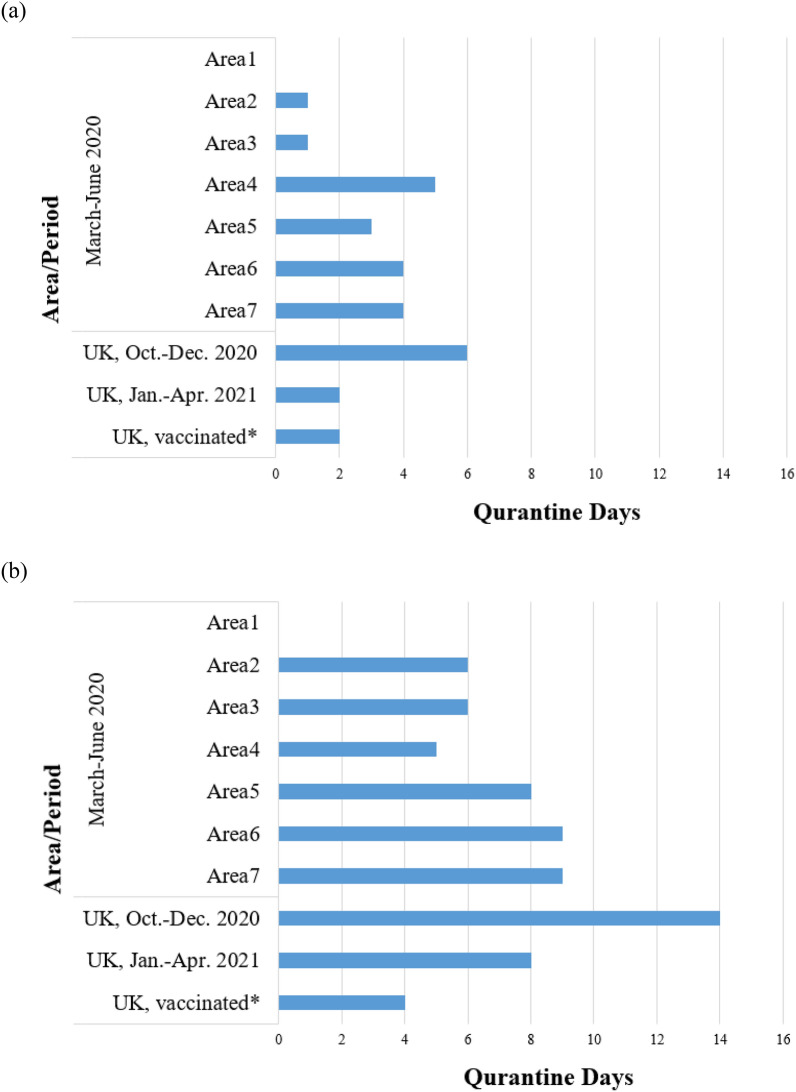
Precision quarantine days for RT-PCR test negative passengers after arrival at airport by the risk of the departing areas and the RT-PCR administrative strategy (**a**) Two RT-PCRs with varying duration of quarantine (**b**) One RT-PCR with varying duration of quarantine; Area 1: Asia; Area 2: Oceania; Area 3: North and South America, excluding the U.S.; Area 4: U.S.; Area 5: Europe, excluding the U.K.; Area 6: U.K.; Area 7: Africa; UK, vaccinated: The vaccine efficacy was applied to the scenario in the UK in October–December 2020.

Figure 2 shows precision strategies of various lengths of quarantine and isolation according to the risk classification of country and region given two or one RTR-PCR test policy. The higher the risk group (Areas 6 and 7 were the highest risk group) was, the longer the day of the scheduled quarantine was. The third period of October–December 2020 required a longer quarantine for the UK even two RT-PCR tests were applied. The vaccination reduced the time required for quarantine in both partial vaccinated in the fourth period. Specifically, the vaccinated travelers in the UK required only two days given one test. It should be noted that screening, quarantine, and isolation on border control can be lifted for the vaccinated travelers if the incidence of pre-symptomatic COVID-19 can be lowered to less than 40 per 100,000 of the country or region where imported cases come from those the vaccine uptake.

## Discussion

We developed a four-compartment stochastic process with Bayesian MCMC algorithm to model a natural history process from uninfected, pre-symptomatic, until symptomatic phase, making allowance for asymptomatic cases, evolving with virus shedding and also the dynamics in the accuracy of RT-PCR test. Based on the two estimated parameters of the daily rate of being pre-symptomatic and the subsequent dwelling time from pre-symptomatic to symptomatic phase, a series of computer simulation experiments were conducted to provide precision border control strategies for inbound passengers.

Specifically, these two applications can be illuminated as follows. First, the daily rate of being pre-symptomatic can be used to stratify the risk of the underlying country/region. Here, we classified seven areas from the lowest to the highest risk in the first period. This estimate is very informative to monitor the epidemic trend of the evolution of virus variants with time, the effectiveness of NPIs and vaccine. It can be clearly seen that this estimate increased from 106, dropped to 37, rose again to 141 and 90 in the third and the fourth periods, corresponding to the dominant virus variant of D614G in the first period and the new virus variant of B.1.1.7 (Alpha) and B.1.351 (Beta) in the latter two periods, suggesting a 33% increase in the risk of infection. The comparisons of this estimate between the first pandemic period and the second containment period revealed 65% effectiveness of NPIs. The comparison between the vaccinated traveler and the overall risk in the third period suggest 70% effectiveness of vaccine at the start of the phase 4 post-market surveillance. It can be further demonstrated with a 36% effectiveness of vaccination covering around half of passengers from the UK in the fourth period. The second contribution of this study is to provide an insight into the development of the optimal length (day) of quarantine and isolation. The proportion of turning pre-symptomatic to symptomatic cases was up to 80% after five days of self-quarantine and -isolation, and reached to almost 100% after 14 days. Based on these estimates, the time required for quarantine after the administration of RT-PCR test can be reduced from 14 to 5 days as none of COVID-19 cases would be found when two tests with 95% and 80% sensitivity of RT-PCR for the first and the second are offered. The negligible COVID-19 cases would be missed even only one RT-PCR test is administered. Information provided for the vaccinated traveler makes even important contribution to shortening the time required for quarantine into two days while vaccine is distributed but has yet reached the full implementation. More importantly, testing and quarantine measures on border control can be lifted if the risk of pre-symptomatic case is lowered after vaccination. Our estimated results not only provided a refined risk classification by country/region but also proposed a precise scheduled quarantine and isolation with and without considering RT-PCR test as shown in Supplemental Fig. S1.

This is the first study, to the best of our knowledge, to fit the proposed four-compartment stochastic natural history model of COVID-19 with Bayesian MCMC algorithm to empirical data on imported cases, yielding the empirical estimates of the daily risk of being pre-symptomatic and the subsequent progression rate from pre-symptomatic to symptomatic phase together with risk of being asymptomatic through RT-PCT test for close contact with symptomatic imported cases. The median dwelling time from a pre-symptomatic to symptomatic phase was estimated as 3.4 days in the first period, which is longer than the 2.3 days in an early outbreak in Wuhan, China^16^. The disease progression rate might vary from country to country, which possibly depends on virus shedding. Since Taiwan is a very low infection area with very few identified COVID-19 cases the government policy for the identification of pre-symptomatic imported COVID-19 cases when they had passed through airport resorts to 14-day self-quarantine and self-isolation until they turned to symptomatic cases. The empirical data on Taiwanese imported COVID-19 cases provide a natural opportunity to model the natural history of COVID-19 from different countries and regions. We also estimated the proportion of asymptomatic cases based on empirical data from Taiwan. The estimated 16.7% of asymptomatic cases was close to a recent meta-analysis (17%) in the same first period of our study^6^. However, it can be seen that the proportion of asymptomatic cases increased in the latter periods, which may be attributed to the frequent application with RT-PCR test and emerging viral variants. As mentioned in the method section, the natural history model also provides the equivalent control group as used in the randomized controlled trial without being affected by RT-PCR test, namely 14-d Q as shown in Table 2, for the comparison of a series of intervention groups with RT-PCR test and precise intervals for quarantine and isolation. It should be noted that the main advantage of using RT-PCR test as opposed to self-quarantine and self-isolation with 14-day is to facilitate the early detection of pre-symptomatic and asymptomatic COVID-19. The former may be re-captured until they turned to symptomatic cases during 14-day self-quarantine and self-isolation but the latter has to be identified through RT-PCR test. Although the viral variants emerged in the latter period and the sensitivity of antigen tests may be reduced, those pre-symptomatic and asymptomatic COVID-19 cases with high viral load still can be detected by RT-PCR test^17–19^. In addition to our study, a recent study conducted by Dicken et al.^20^ has used a mathematic formula to evaluate the impact of border control and quarantine measures based on the imported and secondary cases from different epidemic regions. However, their proposed method may not be specified for targeting pre-symptomatic COVID-19 cases as their method did not separate pre-symptomatic cases from asymptomatic cases. Their study focused on the first pandemic period but our study covers three periods. The proposed method and results here would not only monitor the epidemic trend evolving with viral variants with time but also provide an opportunity to develop precision strategies for vaccinated and unvaccinated travelers.

**Figure 3:**
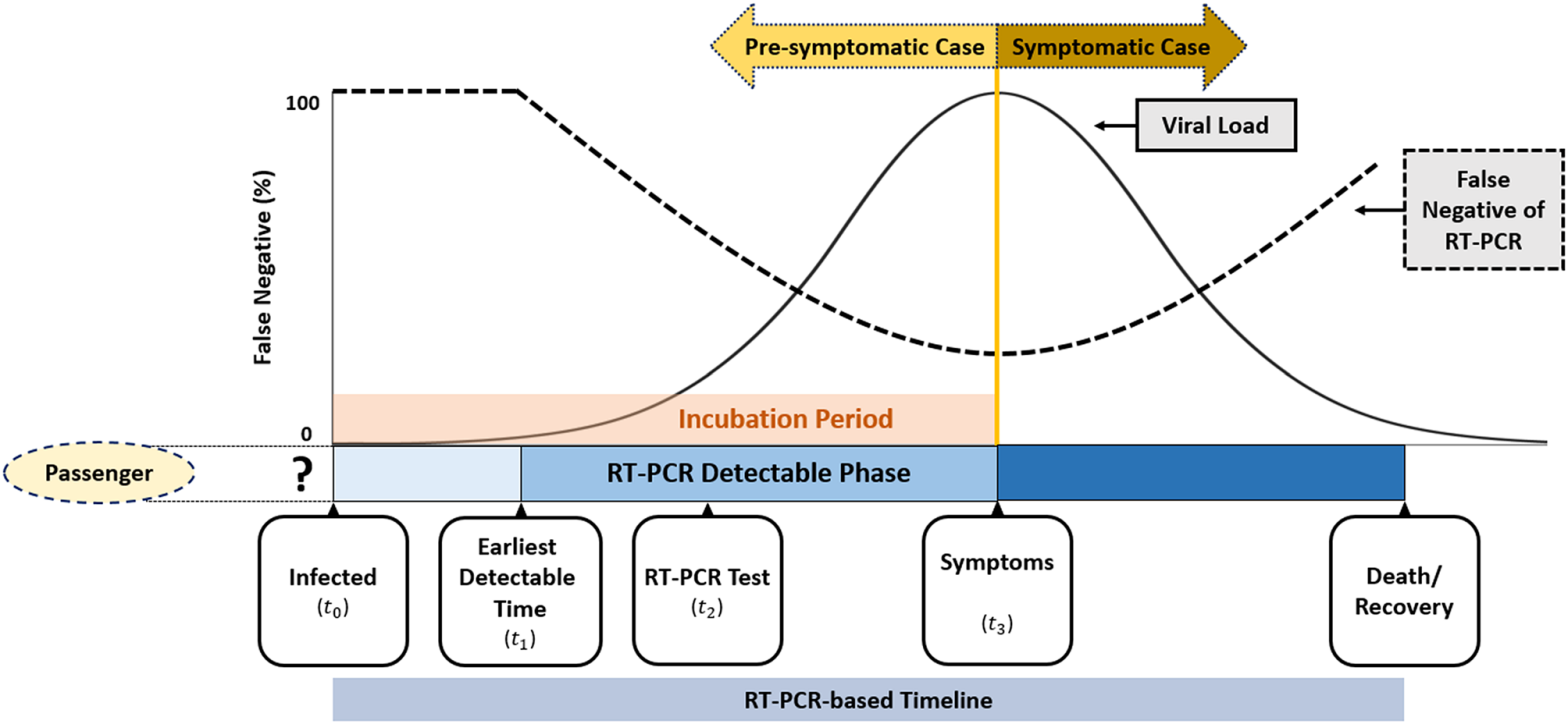
The conceptual diagram for the RT-PCR-based defined disease progression in association with the detectability of the test.

There are limitations in this study. Firstly, as indicated in Fig. 3, our simulation results would be affected by the test sensitivity. In this study, the RT-PCR assays as used for screening with sensitivity varying with time but assuming the quality of laboratory diagnosis is identical across countries. However, the results of identifying COVID-19 cases relying on RT-PCR assays may vary with countries because of other factors related to laboratory diagnosis, including sampling location, improper clinical sampling, the quality of RNA extraction, the sensitivity of the detection kits and the variation of targeted viral RNA sequences^21^. This could be improved by changing the criteria from laboratory diagnosis to clinical diagnosis with more competent computer tomography^22^. However, whether the criteria of clinical diagnosis is appropriate for border control deserves a further research. Secondly, the current results in this study can only be applied to the known variants of concern (VOC) encountered in this study, such as D614G and VOC Alpha. With varying characteristics of each VOC, a substantial modification of the solutions proposed in this study has to be considered.

In summary, we developed a pre-symptomatic incubation model governing the occurrence and the progression of pre-symptomatic COVID-19 cases on which we are based to design precision containment in border control of imported cases with the optimal frequencies of RT-PCR test and the optimal interval of quarantine and isolation. The findings for vaccinated and unvaccinated travelers have significant implications for resuming pre-pandemic social and economic activity when facing the imported COVID viral variants and the uneven distribution of vaccine globally.

## Materials and methods

### The pre-symptomatic incubation COVID-19 model

In order to estimate the occurrence and the dwelling time of the imported pre-symptomatic COVID-19 through RT-PCR test or self-quarantine, we here build up a novel natural history model for depicting the dynamic process of being pre-symptomatic first and then surfacing to symptomatic phase as shown in Fig. 3 instead of following the conventional infectious process of COVID-19 from infected time, time to become infectious, the disease onset of showing symptoms although both are correlated through the evolution of viral load from pre-symptomatic to symptomatic phase. The reason of introducing RT-PCR detectable phase from the earliest detectable time to the presence of symptoms is that pre-symptomatic COVID-19 can only be detected by RT-PCR until the viral load rises to the detectable threshold. Accordingly, there are two main clinical parameters of interest in such a dynamic natural course, the daily rate of being pre-symptomatic and the mean dwelling time between pre-symptomatic and symptomatic phase during incubation period. The sensitivity of RT-PCR may also vary with the evolution of disease progression and the viral load. Note that asymptomatic cases would be defined when the dwelling time is infinite when no RT-PCR test is administered.

Following the Fig. 3, all imported cases of COVID-19 are classified into three types, symptomatic, pre-symptomatic, and asymptomatic cases (Supplementary Fig. S2). The symptomatic cases were defined for those reporting symptoms upon arrival. Those who had no symptoms when arriving at the airport, but developed symptoms during self-quarantine, were classified as pre-symptomatic cases. The asymptomatic cases included those who have never developed symptoms but were confirmed with RT-PCR examinations in the period of quarantine because of close contact with symptomatic COVID-19 cases.

### Containment measures for COVID-19 in Taiwan

Since 2004, all airports in Taiwan have been equipped with infrared temperature measuring instruments to screen and identify passengers with fever in response to the outbreak of SARS occurring in 2003^23,24^. After the confirmed cases of COVID-19 surged up in Wuhan in early January, Taiwan Centers of Disease Control listed SARS-CoV-2 as a category 5 among all communicable diseases on 15th January, which also includes Middle East respiratory syndrome coronavirus (MERS-CoV), H7N9 influenza, Ebola virus disease, and yellow fever. The Central Epidemic Command Center (CECC) was established on 20th January to integrate the resource of the administration sectors and hold daily press for reporting the epidemic profiles of COVID-19 cases.

There are several decisive time points for border control in an attempt to contain the community-acquired outbreak of COVID-19 in Taiwan ^25–27^. On 7th February, Taiwanese government launched a border control to stop foreign travelers, who had visited or resided in mainland China, Hong Kong and Macao within 14 days, from entering Taiwan. Since then, all Taiwanese residents coming back from mainland China followed a 14-day home quarantine. For those having shown symptoms upon arrival, they would receive an instant RT-PCR test at airport and be sent to hospitals for isolation. Only those with two consecutive negative results (24 h apart) can be released and go home, but they were still required to have another 14-day home quarantine. Those with a RT-PCT positive result should stay at hospitals and can only be discharged after three consecutive negative tests. The close contacts of the confirmed cases were also requested to have a 14-day home quarantine.

Following the surge of confirmed cases in Europe and in the U.S. in early March, the CECC further announced the border control, quarantine and isolation measures on 14th March to all passengers who travelled back from areas with high epidemic risks in addition to China. An advanced travel ban, which rejected all foreign nationals from entering Taiwan as of 19th March onward, was issued. All Taiwanese entering Taiwan had to also follow the 14-day home quarantine measure. In addition, the CECC commended a retrospective health monitoring on those with a travel history within 14 days in Europe and in the U.S. They were required to contact local health authorities and those with symptoms must be tested. Note that such a travel ban would affect the estimation of parameters in relation to the disease natural history of imported cases from different countries in different periods.

### Data sources

To elucidate the abovementioned natural history of COVID-19, we targeted the imported cases of COVID-19 among inbound passengers around the world who flew into Taiwan between March 2020 and April 2021 and excluded domestic cases infected in Taiwan as the latter may also include the unknown origin of contact history that may preclude us from estimating the relevant parameters of natural history. For each confirmed case, data were retrieved from a repository that has summarized information on imported cases reported by the CECC in Taiwan^28^. Information on the time stamped on the date of arrival and departure from foreign countries, date of arrival at Taiwan, date of the occurrence of clinical symptoms, and date of RT-PCR test were abstracted from the CECC press. Note that the starting date and time at point of entry to Taiwan consist of two categories, departure from and arrival to Taiwan for Taiwanese citizenship and departure from the disembarked country and the arrival to Taiwan for foreigners. Following the guideline of Taiwan CECC, for a subject with suspected symptoms of COVID-19 including fever, cough, short of breath, fatigue, myalgia, diarrhea, and anomaly in smell and taste, they would be tested for SARS-CoV-2 infection with RT-PCR twice within 24 h. For inbound passengers without symptoms, it is mandatory for these subjects to be under quarantine for 14 days. The quarantined subjects were tested for SARS-CoV-2 upon the occurrence of suspected symptoms during their 14-day quarantine^28^. Information on the origin of country/region for these inbound cases were also collected. Supported by the empirical data of time to show symptoms of COVID-19 upon entry and during 14-day quarantine period, we estimated the daily rates of being pre-symptomatic as well as the dwelling time from pre-symptomatic to symptomatic phase. Date on the number of passengers arriving at Taiwan with information on the departure countries were retrieved from the open data source of the Ministry of Transportation and Communications, Taiwan^29^ and Ministry of the Interior National Immigration Agency, Taiwan^30^. All methods were carried out in accordance with relevant guidelines and regulations.

### Bayesian MCMC algorithm for estimating parameters

To estimate the daily rate of being pre-symptomatic and asymptomatic and the dwelling time from pre-symptomatic to symptomatic phase, we applied a four-state stochastic model to quantify the disease progression from uninfected (State 1), through pre-symptomatic phase (State 2), and finally to symptomatic phase (State 3) or still remained asymptomatic phase (State 4) in the light of the previously established multi-state stochastic model^31–33^ (Supplementary Fig. S3). The daily rate of being pre-symptomatic COVID-19 provides the risk stratification of the country/region where imported COVID-19 cases travelled from. The dwelling time plays a crucial role in the determination of the optimal length of self-quarantine and self-isolation and also the time required for the scheduled quarantine and isolation. Given the evidence that viral shedding changed over time since exposure^34^, we applied the log-logistic function to estimating the transition rate from pre-symptomatic to symptomatic phase for the possible inverse U-shape of the contagiousness of SARS-CoV-2. Bayesian MCMC algorithm was used to estimate the point estimate and 95% credible interval of the relevant parameters in the light of the posterior distribution that was formed by non-informative prior and the likelihood function supported by the empirical data on different subtypes of imported cases as shown in Supplementary Fig. S2. The mathematical likelihood function forms refer to the previous study on the application of the stochastic model^35–37^. Note that the asymptomatic cases detected by RT-PCR test would be analogous to over-detected cases if the screening is offered as asymptomatic COVID-19 like over-detected cases in screening would have not been found had the RT-PCR screening been not administered. In order to make the asymptomatic proportion representative of the underlying imported cases instead of being limited to the contacts, we incorporated the proportion of asymptomatic cases in some particular groups (such as the member in a travelling group with more than 2 confirmed COVID-19 cases) for whom the RT-PCR tests had been fully offered at the point of entry to re-weight the part of the likelihood function contributed from asymptomatic cases. In order to consider the effects of the infected source of the country and the period for each inbound passenger, we applied the exponential regression models with the following regression equations *λ*_*i*_ = *λ*_*i*0_ × exp(βX); where *λ*_*i*_ and *λ*_*i*0_ represent the three parameters of transition rates and the corresponding baseline rates; X and β represent country/region and period and their corresponding regression coefficients. The program was written by using the MCMC procedure of SAS version 9.4.

### The algorithm for computer simulation experiments

In order to provide the precision border control strategies by integrating RT-PCR tests together with an optimal quarantine period for containing the transmission of SARS-CoV-2 resulting from imported cases, a three-arm computer simulation experiments for the eligible population of incoming passengers with different statuses of COVID-19 (uninfected, asymptomatic, pre-symptomatic, and symptomatic phase) was designed. The details are delineated in Supplementary Fig. S1 with various scenarios.

### Three border control strategies

1. RT-PCR test on arrival and quarantine for five days for subjects with negative initial results followed by a second RT-PCR test at the end of quarantine (Arm1: Two RT-PCRs + 5-d Q, Supplementary Fig. S1),
2. RT-PCR test on arrival and quarantine for five days (Arm2: One RT-PCR + 5d Q, Supplementary Fig. S1), and
3. quarantine for 14 days without test (Arm3: 14-d Q, Supplementary Fig. S1).

A shortened quarantine period of 5 days was first applied according to the time required from pre-symptomatic to symptomatic phase estimated from the four-state stochastic process in conjunction with empirical data on imported cases. To explore the possible impact of a longer quarantine, a 7-day period for Arm 1 and Arm 2 were also assessed. The rationale of comparing these three arms is that while RT-PCR test can detect subjects with asymptomatic and pre-symptomatic COVID-19, passengers surfacing to symptomatic COVID-19 would also be identified during the quarantine period, which is the main rationale of using only quarantine as a border control measure. In the meantime, the strategy of quarantine alone would miss passengers who would not present symptoms and signs.

To take into account the impact of RT-PCR accuracy on these missed COVID-19 cases and the possibility of a lower sensitivity for the second test, 95% and 80% sensitivity for the first and the second test were applied^38,39^. In addition, the strategies with the quarantine period of 5, 7, and 14 days were also considered for Arm1 and Arm2.

### Periods of COVID-19 pandemic with vaccinated travelers

Moreover, since our study covers four periods the computer simulation experiments were applied to the third and the fourth pandemic period but only limited to the United States of America (USA) and the United Kingdom (UK) as the viral variants in both regions were remarkably noted after October 2020. Most importantly, the simulated design was also applied to the vaccinated traveler based on the UK scenario in the third period when the vaccine was not available, in contrast to the fourth period when half of UK residents were vaccinated, which was used as an illustration for the preparedness of border control during the period from the launch of vaccination until mass vaccination worldwide. The efficacy of vaccine applied to travelers from the UK was based on the results of phase 2/3 trial for chimpanzee adenovirus Oxford 1 (ChAdOx1) nCoV-19 in the UK^40^. The administration of low dose in the first dose and a standard dose as the second dose of ChAdOx1 nCoV-19 can reduce symptomatic and asymptomatic cases by 90% (67.4–97.0%) and 58.9% (1.0–82.9%), respectively, implying the corresponding relative risk of 0.10 (0.03–0.33) and 0.41 (0.17–0.99). The logarithm transform led to the point and interval estimates of the two regression coefficients. Assuming both follow normal distribution, we have two corresponding regression coefficients followed N(mean = − 2.3076, sd = 0.6055) and N(mean = − 0.8882, sd = 0.4490) to the incidence rates of pre-symptomatic and asymptomatic cases for the vaccinated passenger.

### Country-specific precision strategies

In addition to the presentation of three main strategies, the precision strategies on the days of quarantine and isolation for each country or region given only quarantine and isolation, one test, two tests, and also the vaccinated were estimated on the basis of the criterion of no pre-symptomatic case detected.

### Bayesian MCMC algorithm for computer simulation experiments

A total of 15,000 passengers were randomly assigned into the three arms with 5000 in each arm for any given geographic regions in each period. For subjects assigned to each of the study arms, the area-specific risk of being infected and the daily risk of clinical evolution of COVID-19 in terms of asymptomatic, pre-symptomatic, and further progression to symptomatic COVID-19 were projected by the predictive distribution that was derived from the posterior distribution encoded with the estimated parameters as detailed above. We also used a Bayesian MCMC algorithm with 200,000 repetitions and the thinning period of 20 was applied to deriving 5000 rounds of trials based on the predictive distribution. The inferences on the number of missed COVID-19 cases for each study arm would be drawn on the basis of these samples. The simulation algorithm was performed by using the MCMC procedure in SAS version 9.4.

## Supplementary Information

Supplementary Information.

## Data Availability

All datasets used in this study are available from References^9–11^.
