# Supplementary material for: A pre-symptomatic incubation model for precision strategies of screening, quarantine, and isolation based on imported COVID-19 cases in Taiwan
Source: Sci Rep. 2022 Apr 11;12:6053. doi: 10.1038/s41598-022-09863-w (PMC8998162; doi:10.1038/s41598-022-09863-w)
Supplement: Supplementary file 1 — Supplementary Information. [file 41598_2022_9863_MOESM1_ESM.docx]

**Supplementary Materials for**

**Jen et al, “*A Pre-symptomatic Incubation Model for Precision Strategies of Screening, Quarantine, and Isolation based on Imported COVID-19 Cases in Taiwan*”**

**Supplementary Table S1.** Number of population, confirmed cases of COVID-19, visitor arrivals, imported cases in Taiwan by different areas between March 2020 and April 2021

| Period/Area | Visitors Arrivals | Imported cases | | | |
| --- | --- | --- | --- | --- | --- |
|  |  | **Asymptomatic (%)** | **Pre-symptomatic**  **(%)** | **Symptomatic**  **(%)** | **Total** |
| March-June 2020 | 293,750 | 19  (5.6) | 143  (42.3) | 176  (52.1) | 338* |
| Asia | 236,455 | 5  (9.8) | 21  (41.2) | 25  (49.0) | 51 |
| Ocean | 4,200 | 0  (0.0) | 2  (50.0) | 2  (50.0) | 4 |
| Am, excl. USA | 6,802 | 1  (6.3) | 6  (37.5) | 9  (56.3) | 16 |
| USA | 23,382 | 3  (3.5) | 39  (45.9) | 43  (50.6) | 85 |
| EU, excl. UK | 18,602 | 7  (7.7) | 43  (47.3) | 41  (45.1) | 91 |
| UK | 3,486 | 1  (1.4) | 23  (31.1) | 50  (67.6) | 74 |
| Africa | 823 | 2  (11.8) | 9  (52.9) | 6  (35.3) | 17 |
|  |  |  |  |  |  |
| July- September 2020 | 141,932 | 22  (27.8) | 15  (19.0) | 42  (53.2) | 79 |
| Asia | 117,625 | 19  (30.2) | 10  (15.9) | 34  (54.0) | 63 |
| Ocean | 913 | 0  (0.0) | 0  (0.0) | 1  (100.0) | 1 |
| Am, excl. USA | 1,961 | 0  (0.0) | 0  (0.0) | 3  (100.0) | 3 |
| USA | 9,351 | 1  (25.0) | 2  (50.0) | 1  (25.0) | 4 |
| EU, excl. UK | 9,091 | 2  (66.7) | 0  (0.0) | 1  (33.3) | 3 |
| UK | 2,519 | 0  (-) | 0  (-) | 0  (-) | 0 |
| Africa | 472 | 0  (0.0) | 3  (60.0) | 2  (40.0) | 5 |
|  |  |  |  |  |  |
| October-December 2020 | 145,767 | 220  (70.3) | 55  (17.6) | 38  (12.1) | 313 |
| Asia | 122,329 | 196  (85.2) | 19  (8.3) | 15  (6.5) | 230 |
| Ocean | 949 | 1  (100.0) | 0  (0.0) | 0  (0.0) | 1 |
| Am, excl. USA | 2,214 | 0  (0.0) | 0  (0.0) | 1  (100.0) | 1 |
| USA | 9,712 | 9  (22.0) | 22  (53.7) | 10  (24.4) | 41 |
| EU, excl. UK | 7,801 | 9  (39.1) | 8  (34.8) | 6  (26.1) | 23 |
| UK | 1,974 | 4  (28.6) | 4  (28.6) | 6  (42.9) | 14 |
| Africa | 788 | 1  (33.3) | 2  (66.7) | 0  (0.0) | 3 |
|  |  |  |  |  |  |
| January-April 2021 | 176,004 | 217  (67.6) | 70  (21.8) | 34  (10.6) | 321 |
| Asia | 122,329 | 164  (80.4) | 27  (13.2) | 13  (6.4) | 204 |
| Ocean | 949 | 0 | 0 | 0 | 0 |
| Am, excl. USA | 2,214 | 8  (50.0) | 7  (43.8) | 1  (6.3) | 16 |
| USA | 9,712 | 18  (41.9) | 18  (41.9) | 7  (6.3) | 43 |
| EU, excl. UK | 7,801 | 16  (57.1) | 9  (32.1) | 3  (10.7) | 28 |
| UK | 1,974 | 6  (54.5) | 3  (27.3) | 2  (18.2) | 11 |
| Africa | 788 | 5  (26.3) | 6  (31.6) | 8  (42.1) | 19 |

* 36 imported cases with source countries unknown were excluded.

**Supplementary Table S2.** The estimated daily rate of being pre-symptomatic and asymptomatic and progression from pre-symptomatic to asymptomatic COVID-19 among Taiwanese imported cases between March 2020 and April 2021.

|  | Daily risk of being pre-symptomatic  (per 10^5^)  (95% CI) | Median Time to develop symptoms (Days)  (95% CI) | Proportion of Asymptomatic case (%)  (95% CI) |
| --- | --- | --- | --- |
| Asia | 18.0  (13.1-24.3) | 3.49  (2.71-4.38) | 21.2  (11.4-33.9) |
| Oceania | 79.5  (22.1-188.0) | 5.65  (2.71-10.26) | 4.7  (0-30.2) |
| Am, excl. USA | 188  (98-309) | 4.22  (2.22-7.08) | 8.4  (0.7-27.4) |
| USA | 339  (271-411) | 3.28  (2.67-3.97) | 13.3  (6.7-21.6) |
| EU, excl. UK | 414  (343-495) | 4.05  (3.37-4.88) | 24.8  (17.8-32.1) |
| UK | 1110  (882-1370) | 3.26  (2.33-4.33) | 7.0  (2.9-13.1) |
| Africa | 1400  (806-2310) | 2.99  (2.00-4.33) | 9.7  (0.5-29.3) |

**Supplementary Fig. S1.** The computer simulation three-arm experimental design.


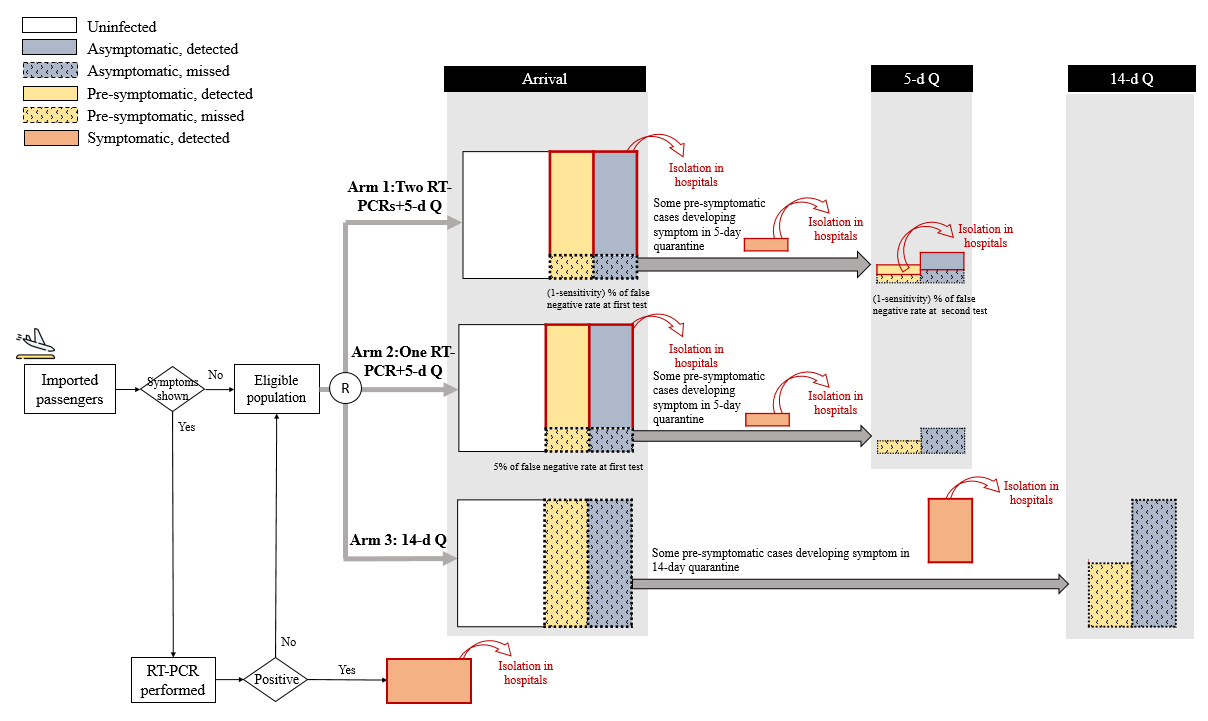


**Supplementary Fig. S2.** Data display for the classification of the subtypes of COVID-19 cases.


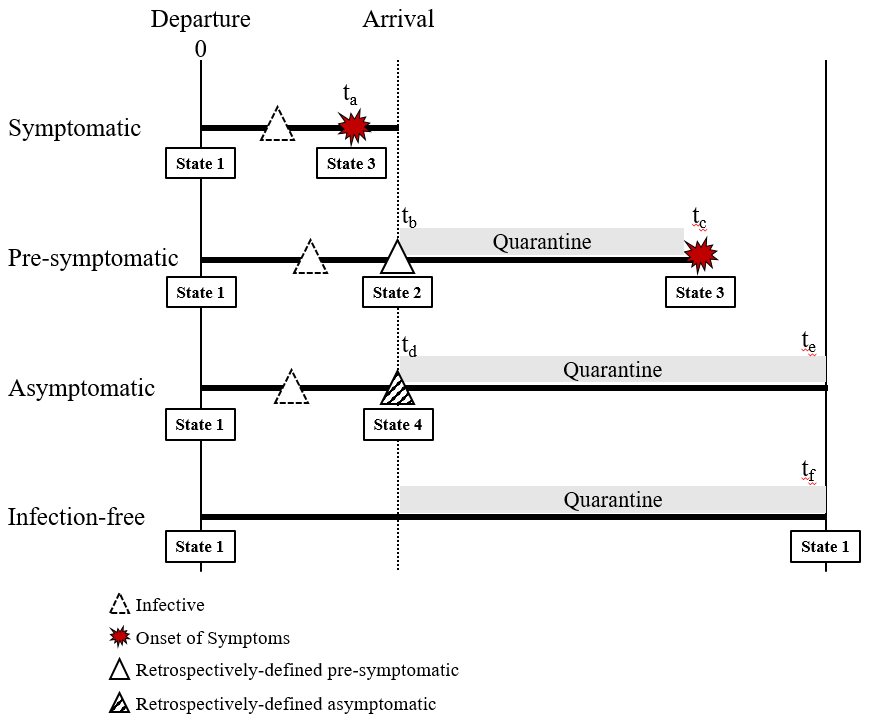


**Supplementary Fig. S3.** The four-compartment stochastic model for the natural history of COVID-19

Uninfected

(State 1)

Symptomatic phase

(State 3)

Pre-symptomatic phase

(State 2)

Asymptomatic phase

(State 4)
